# Role of selective attention in fatigue in neurological disorders

**DOI:** 10.1111/ene.15739

**Published:** 2023-02-24

**Authors:** Annapoorna Kuppuswamy

**Affiliations:** ^1^ Queen Square Institute of Neurology University College London London UK

**Keywords:** attention, fatigue, sensory attenuation

## Abstract

**Background:**

Chronic fatigue is a significant symptom in several diseases including traumatic and degenerative neurological disorders. While several studies have investigated the correlates of chronic fatigue, there is as yet no unifying framework to explain chronic fatigue.

**Methods:**

In this narrative review, I investigate the role of selective attention in the development of chronic fatigue and discuss results within the framework of the sensory attenuation model of fatigue, which posits that fatigue is the phenomenological output of altered attention to sensory input. Following a short introduction of this framework, I present results from investigations that address attentional mechanisms in fatigue in multiple sclerosis, stroke, traumatic brain injury and Parkinson's disease.

**Results:**

Attention was quantified in all four disease models using a variety of outcome measures, including behavioural, neurophysiological, structural and functional brain connectivity. The range of measures precluded direct comparison of results across disease conditions; however, in all four disease models there was evidence of poor selective attention that explained levels of chronic fatigue, supporting the sensory attenuation model of fatigue as a disease‐independent mechanism of fatigue. Evidence was lacking to draw any conclusions about the direction of causality.

**Conclusion:**

The role of selective attention in development of fatigue is indicated. Future studies must focus on establishing causality and exploring attentional circuitry as a potential therapeutic target.

## INTRODUCTION

Chronic diseases present with fatigue as a significant symptom. Despite high prevalence, a definitive cause of chronic fatigue is still uncertain. While numerous studies investigate the correlates of fatigue [1, 2, 3], a causal link is difficult to determine, given the multifactorial nature of fatigue and, more importantly, the dual relationship between fatigue and any associated variable of interest. For example, reduced activity can both result in, and be result of, fatigue. Therefore, to identify a causal link between fatigue and activity it is critical to know how reduction in activity results in fatigue. In this view, a robust theoretical framework that explains how fatigue is generated is imperative. Here, I examine the evidence linking selective attention to fatigue in four neurological disorders – stroke, multiple sclerosis (MS), Parkinson's disease (PD) and traumatic brain injury (TBI) – and explain how changes in selective attention might be causally linked to fatigue using the sensory attenuation framework of fatigue [4, 5].

At a given moment in time, in an awake human, the brain is confronted with several streams of sensory information, primarily visual, auditory and proprioceptive. Only a small percentage of this information is actively processed by the brain. The sensory information that is processed is partly dependent on goals and intentions, and partly determined by hard‐wired mechanisms designed to distinguish between self‐generated and externally generated stimuli. In the case of proprioceptive and somatosensory information, self‐generated sensory input such as stretch of the skin due to movement is always attenuated, thought to be fundamental to the sense of volition attributed to voluntary movement [6]. In the case of visual and auditory input, goals and intentions determine which inputs are processed and which are attenuated [7]. The sensory attenuation theory of fatigue [5] posits that (i) fatigue is an inference of task‐related high perceived effort, both predictive (at rest) and during action, (ii) perceived effort is the phenomenological correlate of sensory input gain and (iii) high sensory input gain, or reduced sensory attenuation, is the basis of high fatigue. As regards visual and auditory input, reduced sensory attenuation refers to poor attenuation of non‐target distractor stimuli, commonly reported by those who suffer from chronic fatigue as sensory overload when present in crowded places, and is a significant trigger for fatigue [4]. Distractor suppression is fundamental to selective attention [8]. When distractors are not adequately suppressed, there is greater effort required to selectively attend to the target stimulus. Therefore, poor selective attention can be viewed as being causally linked to fatigue within the sensory attenuation framework of fatigue.

## MULTIPLE SCLEROSIS

A role for attention in MS fatigue has been investigated using both behavioural measures and measures of neural activity and connectivity. A general reduction in response speed [9] and delayed latency of responses is seen in high fatigue [10, 11, 12]. In tests of sustained attention, reduced speed is further exacerbated by repeated performance of the sustained attention task, indicating that self‐reported fatigue influences fatigability in attention tasks [9]. However, a lack of manipulation of attentional load in baseline test conditions makes it difficult to attribute slowed responses solely to attentional deficits. Slowed oral responses may also be driven by overt motor deficits related to speech production or altered sensorimotor processing. Such uncertainties were laid to rest in another study [13] where the difference in response time between a demanding attentional task and a less demanding attentional task was directly related to self‐reported fatigue levels, even after controlling for measures of memory and processing speed. This study used the difference between Trail Making Test B (TMT‐B) and TMT‐A as a measure of task switching. Despite requiring motor capability to write, this confounder was eliminated by using the difference in task responses, with any non‐attentional motor slowing eliminated, as it is the same across the two tasks in a given individual. In the TMT, one is required to identify individual numbers from amongst a group of numbers, in a sequential order, with version B requiring alternating between numbers and letters in a sequential order. While this test has been routinely administered to measure visual search and task‐switching ability, the underlying attentional capacity required to successfully complete the task is to selectively attend to a target stimulus in the presence of distractor stimuli (e.g., identifying number 1 amongst 10 different numbers on the chart). Therefore, this well‐controlled study in a relatively large number of MS patients shows that diminished selective attention is strongly related to high fatigue. Despite a robust link, the direction of causality in this relationship is still unclear. However, there is a hint that such deficits may be driving fatigue based on investigations using mediation analysis which show that deficits in attention and processing speed mediate fatigue, while fatigue does not mediate attentional and processing speed deficits [14].

In high fatigue, performance of attention‐demanding tasks is accompanied by greater neural activation in the bilateral anterior cingulate cortices (ACC), right middle cingulate cortex and left paracentral lobule [15]. With its established role in effort perception, greater activation of ACC in high fatigue is indicative of how cognitive load might be perceived as more demanding despite not necessarily engaging prefrontal attentional networks to any greater levels than those with low fatigue. An alternate explanation could be that the physical task of pressing the button might have been more effortful. Although the hand area of the sensory cortex does not show any greater activation, left paracentral lobule activation suggests increased somatosensory processing, perhaps contributing to greater effort and fatigue. Although task‐related activation does not support greater recruitment of attentional networks, resting state functional connectivity [16] metrics show greater dorsolateral prefrontal cortex (DLPFC)–inferior parietal gyrus connectivity in high fatigue suggesting that attentional networks are in an altered state. While altered resting state in itself does not indicate greater effort perception, the altered state is likely to respond differently during task performance in a manner that is related to greater effort. For example, when the motor cortex is less excitable at rest, greater activation is required to produce an output than if it was more excitable, which is likely more effortful. Further evidence of attentional network involvement in fatigue comes from neurotransmitter receptor‐enriched connectivity analysis of resting state fMRI [17] which shows involvement of noradrenergic circuits, and not dopaminergic and serotonergic networks, with specifically greater connectivity in DLPFC, inferior frontal gyrus (IFG) and cingulate gyrus, further strengthening the role of both attentional and effort awareness networks of the brain in MS fatigue.

## PARKINSON'S DISEASE

In PD the etiopathology is confined to a well‐defined region in the brain, the basal ganglia. It was not until recently that studies into fatigue started broadening their search for underlying mechanisms of fatigue outside of basal ganglia, after it was established that disease severity does not explain fatigue symptoms. In a small but well‐designed study [18], it was shown that bottom‐up attentional mechanisms were significantly more compromised in PD patients with high fatigue than low fatigue, while top‐down attentional mechanisms were equally compromised in all PD patients with and without fatigue when compared to healthy controls. In this study, components of the auditory evoked potential that are unique to novelty of the stimulus (P3a), a marker of bottom‐up attention, was more delayed and smaller in high fatigue, while those components associated with anticipation of stimuli (P3b), a marker of top‐down attention, were similar across all patients. A compromise in how unexpected stimuli are processed has significant implications for selective attention, with such a compromise resulting in poor ability to selectively attend to target stimuli presented amongst distractor (novel) stimuli. This finding supports the idea of poor selective attention as a potential contributor to fatigue in PD. Not only do findings in task‐related neural activity support attentional compromises, but abnormalities in attentional network activity are seen at rest too. Using the metric of ‘Amplitude of Low Frequency Fluctuation’, a marker of spontaneous neural activity, this study showed a fatigue‐related increase in mid‐cingulate and left anterior insula cortical activity and a decrease in right DLPFC activation [19], indicating a greater awareness of effort but suppressed activity in the attentional network hub. Further, using fatigue‐associated abnormally active regions as seed regions of interest, this study showed an increased functional connectivity of all three seed regions, with both ventral attentional network (inferior frontal gyrus) and sensorimotor processing regions (right supplementary motor area [SMA] and pre‐central gyrus), including multisensory integration areas (superior temporal cortex). While this evidence strengthens the role of attentional networks in PD fatigue, it also indicates a significant role for sensorimotor processing, a detailed discussion of which is beyond the scope of this review but can be found elsewhere [4].

Another interesting line of evidence which speaks to the role of attention in fatigue comes from a study which compared the deterioration in performance in a cued versus uncued finger‐tapping task [20]. The researchers showed that those individuals with fatigue deteriorated much faster in the cued task when compared to those without fatigue, while in the uncued task the rate of deterioration was similar. In the cued task, the involvement of attentional networks, especially those that attend to external stimuli (novel possibly) is weakened, and informs both fatigue, and influences task performance.

## TRAUMATIC BRAIN INJURY

Despite fatigue being a significant problem in TBI, there have been very few well‐designed studies tackling the mechanisms of fatigue. A particular problem that appears to be more prevalent in this literature than in other conditions is the use of healthy humans as the control population, largely based on the assumption that all TBI patients have comparable levels of high fatigue. While studies in other neurological conditions presented in this review have a healthy control group, I have only discussed fatigue‐relevant results from within the disease cohort, and not differences between the disease cohort and healthy controls. A key issue with using healthy controls is that the healthy brain is in a different state of equilibrium when compared to an injured brain. Any differences between a healthy and injured brain does not necessarily reflect a pathological outcome and may well reflect the altered state of equilibrium [21]. To truly identify fatigue‐related differences, one must compare those with varying levels of fatigue within the TBI cohort. Using this criterium, two studies were identified which explored fatigue‐related attentional behaviour, neither of which measured brain activity.

In a study which specifically investigated selective attention [22] there was no relationship between reaction times and baseline fatigue levels, although the deterioration in performance over time as measured by reaction times was influenced by fatigue levels. This suggests that behavioural outcome at baseline was not modulated by fatigue, but fatigue certainly influenced fatigability. The task involved sequential number presentation with a requirement of response in the form of button press every time the target number appeared amongst the sequential stream of numbers. In addition to measuring reaction times, a self‐reported measure of ‘effort’ was also collected both in the early and late trials of the task. Interestingly, although reaction times were not related to fatigue, the perception of effort associated with task performance was positively correlated with fatigue at baseline, indicating that despite good performance it was performed with difficulty, also perhaps influencing fatigability. Had brain activity been measured during this task it would have revealed greater processing of distractors as seen in post‐stroke fatigue (see later sections). Another study which performed a more comprehensive battery of neuropsychological tests including divided attention, selective attention, working memory and information processing speed tasks showed that the only variable that explained fatigue levels was the complex selective attention task [23]. The task was similar to that performed in the previously discussed study, with the added difficulty of having to respond to more than one criterion of not just the target digit/alphabet, but attention also needing to be paid to the right colour of the target, something akin to a combined Stroop and selective attention task. Overall, despite the number of studies being small, there is greater homogeneity in the tasks and results to confidently assign a significant role for selective attention in TBI fatigue.

## STROKE

Stroke is a highly heterogenous condition with the location of stroke determining the presentation of deficits. Despite such heterogeneity, and lesion‐specific deficits in motor, cognitive, visual, speech and language manifestations, there is surprisingly little influence of stroke location on the prevalence and severity of fatigue [24]. This indicates fatigue may be a network level and not location‐specific dysfunction. Several studies [25, 26, 27, 28, 29] have investigated the relationship between attention and fatigue in stroke with conflicting results, possibly due to the heterogeneity of stroke survivors, and the presence of a more diverse set of mechanisms that underlie fatigue after stroke.

The TMT, as previously described, has been a popular test of choice with no fewer than four studies using it as a measure of complex executive function and attention. The versions of TMT used in the various studies are variable, with the simplest form being TMT‐A with only numbers, with tests B, C and D progressively including alphabets, days of the week and months into the ‘Trail‐mix’. While two studies showed a significant relationship between fatigue and TMT‐A, TMT‐B and TMT‐D tests [27, 28], two other studies failed to replicate these findings [25, 26]. Studies using other tests of attention, some sustained and some selective, also report mixed results. A study that included only those individuals with mild impairments showed a relation between fatigue levels and sustained attention and divided attention [29]. Another study investigating attention using verbal series attention task showed no relationship to fatigue [30]. While all the above studies used a single load condition in the attention task, which makes it difficult to attribute any significant correlations purely to attentional deficits as discussed previously, a more recent study [31] used a composite score of ‘efficiency’ which eliminated other possible explanations for a significant result. In this study, the ‘efficiency’ score was calculated by subtracting the response time in a congruent flanker task from a non‐congruent flanker task, which is a measure of selective attention. The researchers showed greater fatigue related to a bigger difference between non‐congruent and congruent task reaction times, suggesting poorer selective attention in high fatigue.

Studies from my own laboratory have focused on both attention‐related behavioural and neural responses. In a visual perception selective attention task [32] with multiple load conditions, that required participants to respond to a target stimulus presented within a stream of non‐target stimuli, there was a positive relationship between fatigue levels and response times. However, this relationship did not interact with attentional load, suggesting that the slowed responses were not a measure of attention but were possibly generalised motor slowing. The task was performed in the presence or absence of peripheral distractor stimuli. Behavioural responses were not modulated by peripheral distractors. Using a frequency‐tagged electroencephalography (EEG) technique, we also measured the processing of distractor stimuli and showed that with increasing load there was lower suppression of distractors in those individuals with higher fatigue. These results indicate that performance may not necessarily be compromised in fatigue but that the experience of fatigue arises from the inability to suppress distractor processing. In another study using an auditory attentional task [33] we probed the effect of distractors on the processing of anticipated (top‐down attention) versus novel (bottom‐up attention) stimuli. We showed, as seen in the visual perceptual task, that behavioural measures were unrelated to fatigue in the auditory attentional task; however, the effect of distractors on auditory evoked potentials only to novel stimuli, but not anticipated stimuli, was significantly larger in the high than low fatigue groups. This indicates distractors have a bigger effect on bottom‐up than top‐down selective attention similar to that seen in PD fatigue. Despite findings in the attentional network functioning, a study investigating structural disconnections based on lesion location did not find any systematic relationship between disconnection in particular networks and fatigue levels [34].

## SUMMARY

Fatigue is a highly prevalent problem in several neurological diseases, yet hypothesis‐driven approaches to understanding fatigue have been few and far between. The sensory attenuation framework of fatigue provides a robust theoretical grounding that explains the experience of fatigue in a disease‐independent manner. Diminished attenuation of sensory inputs such as visual and auditory inputs manifest as poor selective attention. As a ‘test’ of this hypothesis, a review of studies in fatigue across both neurodegenerative diseases and acquired brain injury shows, despite some conflicting evidence, a common deficiency in selective attention and poor distractor suppression as robust explanatory factors for chronic fatigue. There is, however, very little evidence to establish the direction of causality. Future studies must focus on modulating selective attention and distractor suppression to probe the nature of its relationship to fatigue. Further, there is cause to believe such brain‐based mechanisms may also underlie chronic fatigue of non‐neurological origin such as in peripheral inflammatory conditions. One such example is ankylosing spondylitis where fatigue is related to reduced gray matter volume in attentional networks [35]. This raises an important question of what the role of the primary pathology is in the experience of fatigue. Once a disease is established, the maintenance of fatigue over time is dissociated from the original trigger, and is almost exclusively dependent on brain‐based mechanisms, thereby making the brain a primary target for effective interventions for chronic fatigue irrespective of the disease. While targeting selective attention may be an effective strategy to combat fatigue, it is also important to note that confounding factors such as sleep disorders may also drive fatigue [36, 37]. In some disorders such as MS, treating sleep disorders can significantly reduce self‐reported fatigue [38]. Future studies must investigate if indeed the brain is the most effective interventional target for reversing chronic fatigue, and the extent to which fatigue‐specific interventions are more effective than targeting potential confounding factors.

## CONFLICT OF INTEREST STATEMENT

There are no conflicts of interest.

## Data Availability

Data sharing is not applicable to this article as no new data were created or analysed in this study.
